# Cavity Lasing of Thioflavin T in the Condensed Phase for Discrimination between Surface Interaction and β-Sheet Groove Binding in Alzheimer-Linked Peptides

**DOI:** 10.1021/acs.jpclett.4c01709

**Published:** 2024-09-12

**Authors:** Piotr Hanczyc

**Affiliations:** † Institute of Experimental Physics, Faculty of Physics, University of Warsaw, Pasteura 5, 02-093 Warsaw, Poland; ‡ Center of Cellular Immunotherapies, Warsaw University of Life Sciences, 02-786 Warsaw, Poland

## Abstract

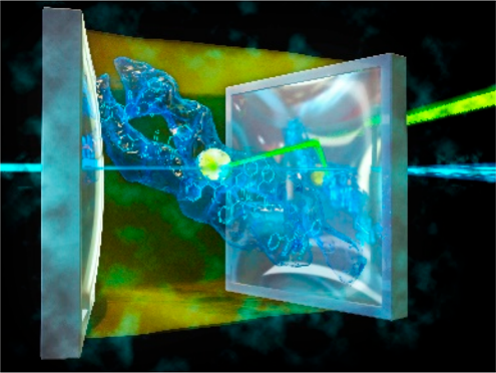

This study investigates the lasing effects in a Fabry–Perot cavity to discern the binding interactions of thioflavin T (ThT) with various peptides associated with Alzheimer’s disease, including Aβ(1–42), KLVFFA, and diphenylalanine (FF) in the condensed phase. Utilizing kinetic lasing measurements, the research explores ThT emission enhancements due to specific groove binding in β-sheet structures and highlights additional contributions from weak surface interactions and solvent–solute interactions. Lasing spectroscopy reveals a lack of transition of the FF system from its native state to an amyloid-like structure, challenging traditional ThT assay interpretations. These findings show the potential of lasing spectroscopy in elucidating the molecular basis of amyloid fibril formation and the development of diagnostic tools for amyloidogenic diseases.

Protein aggregation is a pathogenic process in neurodegenerative diseases, including the most widespread aging disease, which is Alzheimer’s disease (AD).^1,2^ It is reported that, by 2050, one person in every 85 is expected to be diagnosed with AD;^3^ thus, it is an emerging problem to find methods and tools to early diagnose aging diseases before cognitive symptoms occur.^4^ However, the mechanisms by which the protein aggregation contributes to neurodegeneration are not fully elucidated.^5,6^ Supersaturation, a well-known phenomenon in the field of protein crystallization, has also been proposed as an initiation step in protein aggregation.^7^ Applying the concept of supersaturation to amyloid formation suggests that a high concentration of Aβ peptide represents a non-equilibrium state where the peptide concentration exceeds its solubility limit that, in consequence, leads to protein aggregation.^8^

In this letter, a method based on light amplification using a mirror cavity was employed to study the aggregation of Aβ(1–42) and short peptides at high concentrations exceeding the supersaturation conditions, utilizing thioflavin T (ThT) to detect conformational changes and, consequently, ThT binding modes.

Within the Aβ(1–42) peptide sequence, the segment spanning amino acids 16–21, designated as ^16^KLVFFA,^21^ represents a steric zipper core, which is regarded as the essential part for the fibril architecture.^9,10^ This segment inherently forms fibril-like structures characterized by β-sheet grooves. Concurrently, at positions 19–20, a diphenylalanine (FF) is considered as a motif integral to amyloidogenesis.^11^ It is based on the fact that FF self-assembles through the longitudinal stacking of aromatic residues, a mechanism that may also influence the formation of the spine structure of KLVFFA and aggregation of Aβ(1–42).^12^

An argument for considering FF as an amyloid-like structure is supported by staining with ThT, whereby the dye shows fluorescence in microscopy imaging.^13,14^ Owing to these fluorescence characteristics, akin to those observed in amyloid assemblies, self-assembled FF is often posited as the simplest amyloid mimic model. However, despite prevalent assumptions, definitive evidence confirming the amyloid nature of the FF self-assemblies remains ambiguous.

ThT fluorescence is considered as the gold standard for detecting amyloids, primarily because spectral evidence indicates significant enhancement in emission following the formation of a β-sheet structure.^15^ In such configurations, ThT typically lodges within grooves perpendicular to the β-sheet alignment, which prevents the non-radiative twisted intramolecular charge transfer (TICT) state by inducing steric hindrance of the ThT rings, thereby facilitating emission from the locally excited (LE) state.^16^ However, reports suggest that ThT’s association with amyloid aggregates is multifaceted, with possible dye dimerization^17^ and multiple binding modes on a single fibril’s surface.^18,19^

In this study, the lasing effect observed in Fabry–Perot cavities was employed to investigate the amyloid-β peptide Aβ(1–42), its fragment KLVFFA, and the FF motif to study ThT–peptide interactions. The operational principle of the Fabry–Perot cavity lasing technique involves the use of dual mirrors serving as photonic resonators to enhance emitted light within the cavity (Figure 1a).^20^ The setup for measuring lasing is presented in Figure S1 of the Supporting Information. This light amplification initiates once the energy within the cavity surpasses the threshold required for population inversion, leading to the appearance of a distinct lasing peak, as depicted in panels b and c of Figure 1. Beyond this threshold, narrow high-intensity emission peaks appear. The specific pump energy at which this transition occurs is defined as the lasing threshold (Figure 1d).^21^

**Figure 1:**
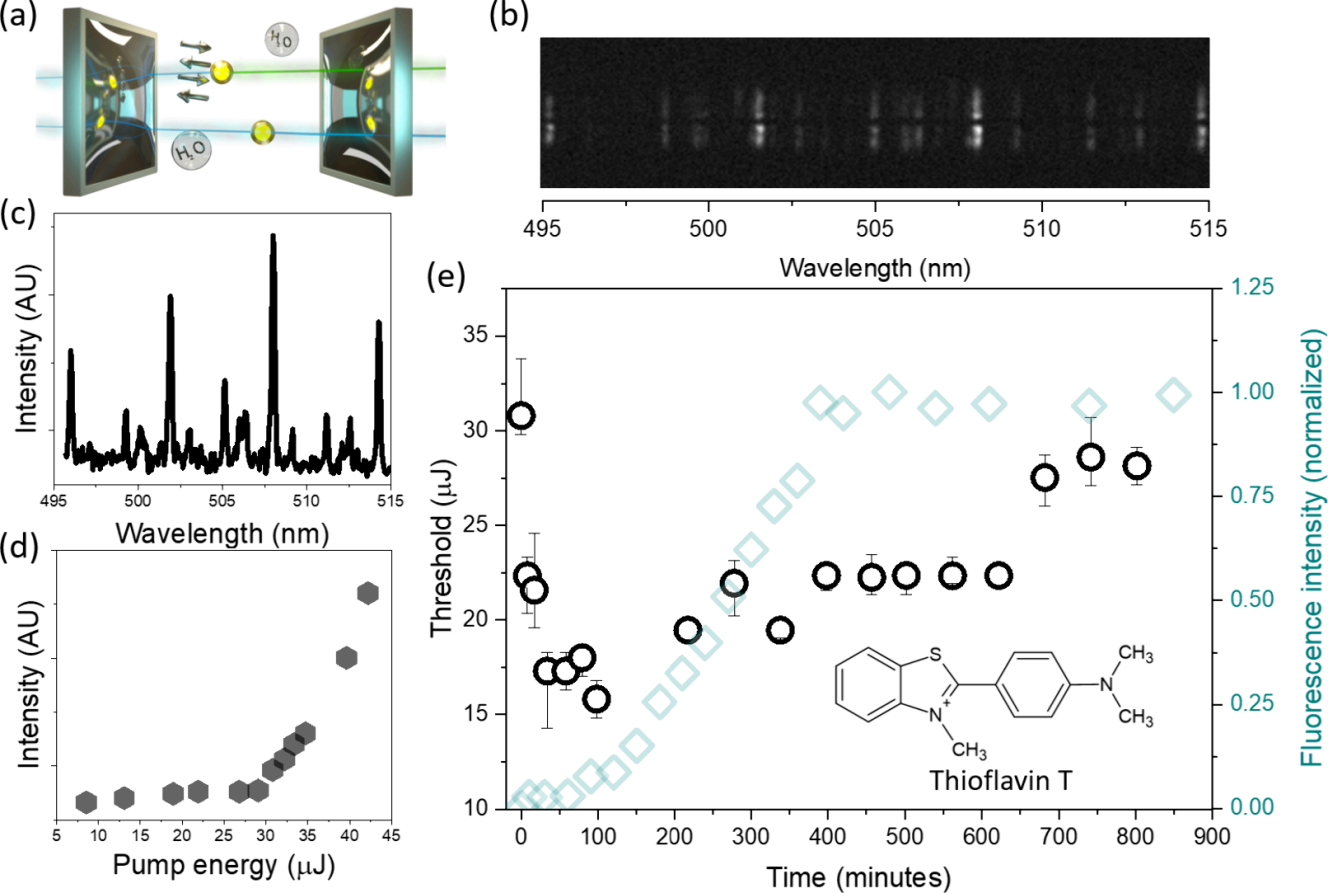
(a) Schematic illustration of a Fabry–Perot cavity containing a liquid medium, which serves as the gain medium, and it contains, dissolved in water, Aβ(1–42) indicated as arrows and ThT dye marked as a yellow dot. The free dot representing ThT dissolved in water shows no light amplification effect. (b) Lasing output from the ThT-doped system, shown as bright spots, indicating successful lasing above threshold levels. (c) Detailed lasing spectrum derived from panel b, featuring multiple peaks within the dye’s emission band. (d) Graph depicting the relationship between pump energy and emitted light intensity, illustrating an exponential increase upon reaching the lasing threshold. (e) Fluorescence assay results for ThT-stained Aβ(1–42) (cyan diamonds), with *C*_ThT_ = 26.1 μM and *C*_Aβ(1–42)_ = 17.2 μM (λ_ex_ = 430 nm), and kinetic analysis of lasing thresholds (black dots), with concentrations of ThT at 26.1 mM and Aβ(1–42) at 17.2 mM (λ_ex_ = 430 nm). Data were collected at set time intervals. The experiment was replicated 3 times, presenting the mean lasing thresholds and associated variability, as indicated by error bars. The inset in panel e shows the chemical structure of ThT.

Figure 1e presents the fluorescence assay and lasing threshold results of ThT-stained Aβ(1–42), which illustrates the temporal relationship between the protein structure and the fluorescence/lasing properties of ThT. In the traditional fluorescence assay, ThT emission is initially negligible, whereas lasing thresholds are identified immediately after mixing the two constituents, ThT and Aβ(1–42). Initial measurements before heating the sample identified lasing thresholds at 31 μJ. A significant reduction in energy required for population inversion was observed upon heating in 37 °C within the first 100 min, reaching a minimum of 15.5 μJ.

After 100 min of incubation ThT and Aβ(1–42) at 37 °C, the fluorescence starts to rise along with the lasing thresholds. The rise of the emission correlates with the formation of protofibrils and the initial development of β-sheet structures. Concurrently, lasing thresholds begin to rise and stabilize at a pump energy of 23 μJ between 200 and 600 min. The formation of mature fibrils leads to an increase in lasing thresholds to approximately 28 μJ.

The initial drop in the lasing threshold within 100 min is likely attributed to the change of the protein conformation due to heating alongside with the change of the microviscosity around ThT molecules. The effects are very weak and do not affect the fluorescence in the typical protein concentration regime, but they are exposed in the lasing experiments in the condensed phase. Later, when Aβ(1–42) tends to form protofibrils, which is associated with rise of emission, ThT binding to the β-sheet grooves becomes evident. The observed increase in lasing thresholds in that phase is likely attributed to the increased scattering of fibrils. As protein aggregation progresses, the resultant aggregates increasingly scatter light, which counteract the fluorescence enhancement.^22^ Consequently, to overcome the scattering effect, more pump energy is required to obtain lasing.

Next, the lasing threshold methodology was utilized to explore the interaction between ThT and FF that has no β-sheet grooves. Modeling studies indicate that specific binding of ThT to the β-sheet grooves of amyloid fibrils necessitates the presence of at least four cross β-strands, creating a surface area of approximately 14 Å, whereas ThT itself spans 15 Å.^23^ Despite the fact that FF is not fulfilling the minimal structural requirements for forming a surface conducive to ThT binding, the presence of FF still results in some fluorescence emission from the dye (Figure 2a).

**Figure 2:**
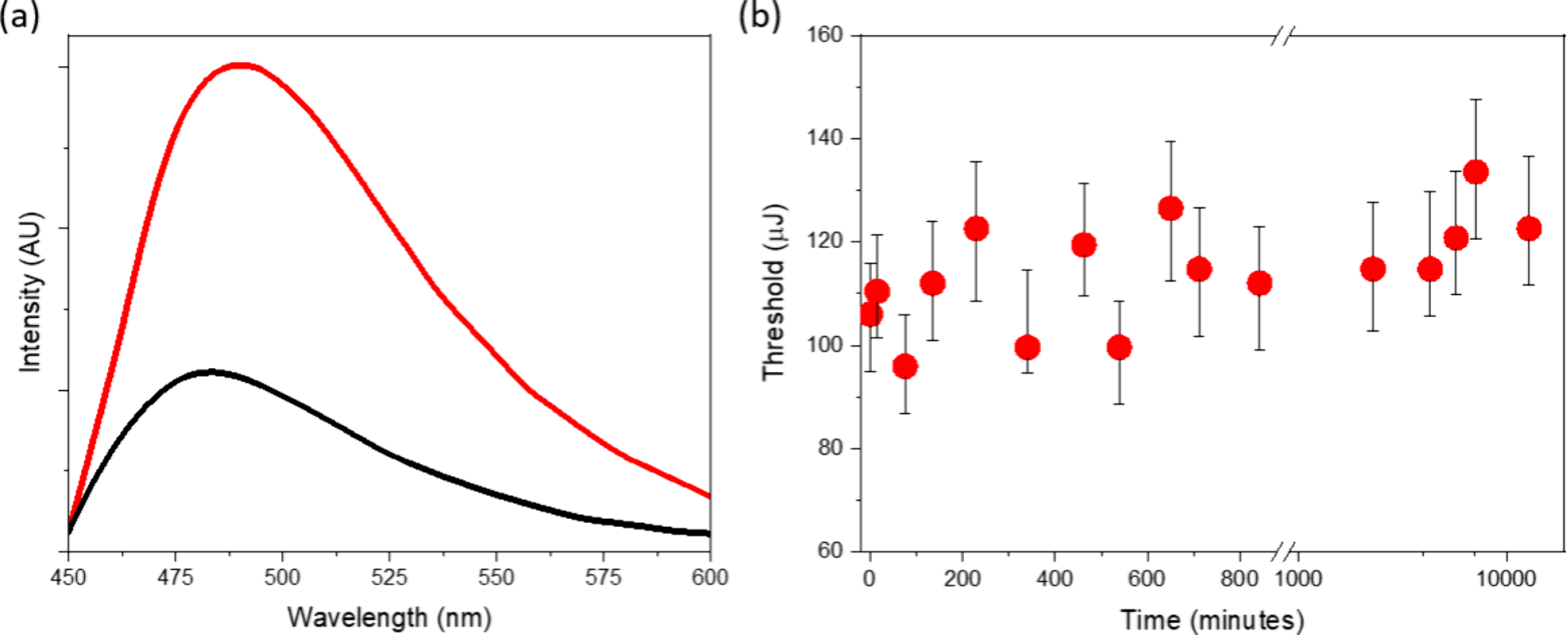
Fluorescence emission spectra of ThT in acetic acid (black) and in the presence of FF dissolved in acetic acid (red), with *C*_ThT_ = 4 μM and *C*_FF_ = 1.6 M. Lasing thresholds measured over time in ThT mixed with FF, with *C*_ThT_ = 3.1 mM and *C*_FF_ = 1.0 M (λ_ex_ = 430 nm).

Figure 2a illustrates the fluorescence characteristics of ThT in the presence of 1.6 M FF dissolved in acetic acid. In comparison to the emission intensity of ThT alone in acetic acid, there is a noticeable increase in fluorescence intensity and a 10 nm red shift in the spectral peak to 490 nm in the presence of FF. These spectral characteristics are reminiscent of β-sheet groove binding, yet the kinetic measurements of lasing thresholds indicate that, unlike in Aβ(1–42), the thresholds do not evolve over time and just fluctuate between the 90 and 120 μJ pump energy range, confirming that there is no conformational change in FF at the molecular level (Figure 2b). A control experiment with fluorescence kinetics confirms no change of fluorescence over time (Figure S2 of the Supporting Information).

This observation implies the existence of an alternative mechanism that is responsible for the enhancement of ThT fluorescence. Wu et al.^23^ have proposed that, in the context of β-sheet grooves, the benzyl ring of ThT, particularly its positively charged nitrogen, is exposed to the solvent. In scenarios involving FF, where no grooves are formed, this exposure is hypothesized to be even more pronounced, as the dye molecule lacks a defined structural niche to bind.

The observation of increased fluorescence of ThT in the presence of high concentrations of FF highlights the role of weak, non-specific interactions, which are significantly augmented by microviscosity in a crowded molecular environment. A similar effect occurs in the presence of high-concentration monomeric proteins and peptides as well as during the early nucleation phase, where ThT emission is primarily responsive to the local microenvironment surrounding the dye molecule.^20^ Fluorescent enhancement in such environment is likely attributed to restricted molecular mobility in densely populated spaces, which facilitates more frequent albeit weak and non-specific interactions among the protein or peptide and ThT molecules (in this particular case between FF and ThT). The fluorescence and lasing results suggest that microenvironmental factors play a crucial role in modulating molecular interactions and their resulting biophysical properties.

The utilization of lasing sensitivity facilitated the distinction between binding to β-sheet surfaces and weak solvent–solute interactions. Panels a and b of Figure 3 depict the temporal measurement of lasing thresholds in ThT mixed with the KLVFFA peptide, dissolved in acetic acid and water, respectively. It is crucial to note that pristine ThT dissolved in water does not produce a lasing effect due to effective TICT, and similarly, no lasing occurs in acetic acid at concentrations of 3.5 mM or lower. This observation aligns with previous studies on lasing in various solvents, likely attributed to the intrinsic viscosity of the solvents, 0.89 × 10^–3^ Pa s for water and 1.12 × 10^–3^ Pa s for acetic acid.^24^ Consequently, all lasing measurements involving peptides in acetic acid were performed with ThT concentrations fixed at 3.1 mM.

**Figure 3:**
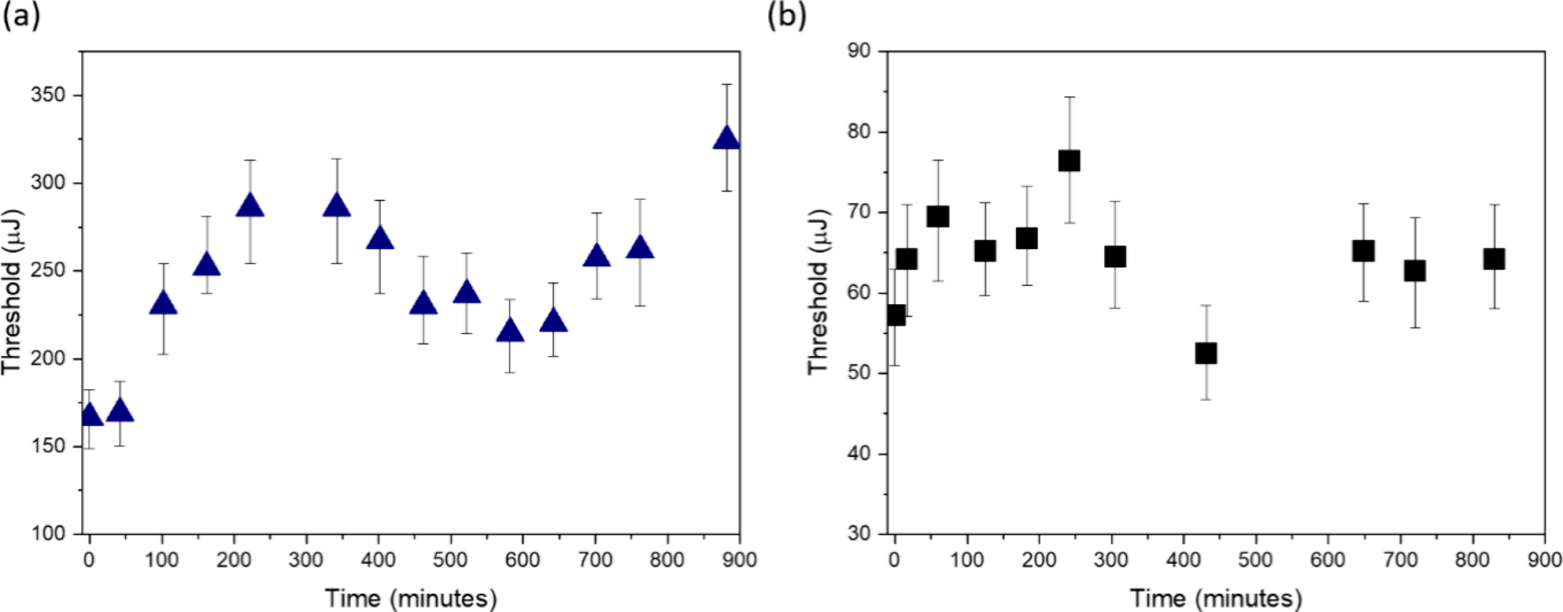
Lasing thresholds of ThT in the presence of (a) KLVFFA dissolved in acetic acid, with *C*_ThT_ = 3.1 mM and *C*_KLVFFA_ = 92 mM, (b) KLVFFA dissolved in water, with *C*_ThT_ = 26.1 mM and *C*_KLVFFA_ = 230 mM (λ_ex_ = 430 nm).

The kinetics of the lasing thresholds measured within 900 min follow the same trend indicating that the KLVFFA aggregation pathway is similar in both solvents. The lasing thresholds for the ThT–KLVFFA conjugate were determined to be between 200 and 300 μJ in acetic acid and between 50 and 80 μJ in water. This discrepancy suggests that solvent macroscopic viscosity is less influential than the microviscosity associated with dye–peptide interactions. Furthermore, the Fabry–Perot cavity used in the experiments had a sample thickness of 13.7 μm in acetic acid and 12.5 μm in water, indicating that the slight variance in the cavity thickness has a marginal impact on the lasing threshold levels.

In control experiments with non-aggregating peptides AAAAAA and GGGGGG, no change in the lasing threshold was observed over 900 min, indicating that molecular crowding is the only driver of the lasing effect in ThT if there is no conformational change in the peptide structure. Another control was done with the LVEAYL peptide, a segment of the insulin protein containing negatively charged glutamic acid (E), which exhibits strong repulsion at high pH.^25^ In an alkali environment, the formation of β-sheet-containing fibrils is inhibited.^26^ Lasing experiments revealed a gradual, linear increase in the lasing threshold, suggesting that, even in a strongly repulsive environment, weak surface interactions between the dye and the peptide surface lead to the light amplification in ThT (Figure S3 of the Supporting Information).

Thus, lasing of ThT observed in β-sheet-forming KLVFFA and non-aggregating peptides confirms that weak surface interactions are significantly enhanced in the condensed phase and can be monitored using lasing thresholds unlike conventional spectroscopic techniques, such as fluorescence, circular dichroism, or infrared spectroscopy, which are insensitive to detect weak surface interactions in the diluted phase.

Lasing results in KLVFFA substantiate that ThT bound to the surface of fibrils is exposed to solvent, with solute–solvent interactions playing a critical role in determining the efficiency of population inversion. Notably, the significantly lower lasing thresholds observed in the water-dissolved conjugate suggest that the angular orientation of ThT may also influence the lasing effects.

Clearly visible fluctuations in the lasing thresholds across both solvents suggest a dynamic shift in the binding mode of ThT. Initially, the effects associated with microviscosity dominate in the early stages of aggregation. However, as the kinetic process of KLVFFA aggregation progresses, there is a notable transition toward β-sheet surface binding. This shift confirms the high sensitivity of ThT lasing, which evolves in response to the structural changes occurring within the aggregating peptide.

In conclusion, the study effectively utilizes the lasing effect within Fabry–Perot cavities to delve into the binding interactions of ThT with peptides associated with AD Aβ(1–42), KLVFFA, and FF motif, highlighting two binding modes: specific binding to β-sheet grooves and non-specific weak interactions. Weak surface interactions were particularly noted in the FF motif, because no transition to amyloid-like structures occurs as no change in lasing thresholds was detected over time. This type of interaction has been previously overlooked in a traditional ThT assay.

Moreover, the study emphasizes the significant role of the microenvironment and solvent–solute interactions in influencing ThT’s lasing thresholds as seen in the case of KLVFFA aggregation in acetic acid and water. Results obtained in the two solvents indicate that subtle changes in the microenvironment, which traditional fluorescence assays might miss, can significantly impact ThT lasing thresholds. This aspect highlights the importance of considering the microviscosity and non-specific interactions at the molecular level, which could lead to a better understanding of the staining properties of ThT and its interaction dynamics with early stage protein aggregates.

This research shows the potential of lasing spectroscopy as a powerful tool for elucidating the molecular basis of protein aggregation formation. This technique proves especially beneficial in developing a lasing-based methodology for amyloidogenic diseases, as it allows for the observation of early stage aggregation dynamics that are often not detectable by conventional methods. This is crucial for advancing the understanding of neurodegenerative diseases, where early detection is critical.

## Supporting Information Available

Materials, preparation protocols, method descriptions, graphical illustration of the setup for lasing experiments, fluorescence spectra of ThT with FF at time 0 and after 24 h, and lasing threshold kinetics of the LVEALYL peptide (PDF)
