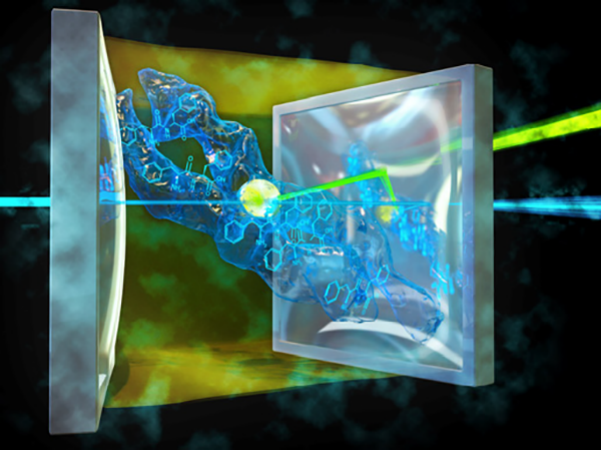# Correction to “Cavity Lasing of Thioflavin T in the Condensed Phase for Discrimination between Surface Interaction and β-Sheet Groove Binding in Alzheimer-Linked Peptides”

**DOI:** 10.1021/acs.jpclett.4c03113

**Published:** 2025-05-05

**Authors:** Piotr Hanczyc

The original TOC image for this manuscript has been replaced with a higher-resolution version.