# Supplementary material for: Cavity Lasing of Thioflavin T in the Condensed Phase for Discrimination between Surface Interaction and β-Sheet Groove Binding in Alzheimer-Linked Peptides
Source: J Phys Chem Lett. 2024 Sep 12;15(37):9543–7. doi: 10.1021/acs.jpclett.4c01709 (PMC11417991; doi:10.1021/acs.jpclett.4c01709)
Supplement: Supplementary file 1 — jz4c01709_si_001.pdf [file jz4c01709_si_001.pdf]

Cavity Lasing of Thioflavin T in the Condensed Phase for Discrimination Between Surface Interaction and β-sheet Groove Binding in Alzheimer-Linked Peptides

Piotr Hanczyc[1,2]*

[1] Institute of Experimental Physics, Faculty of Physics, University of Warsaw, Pasteura 5, 02-093 Warsaw, Poland

[2] Center of Cellular Immunotherapies, Warsaw University of Life Sciences, 02-786 Warsaw, Poland

*Correspondence to: piotr.hanczyc@fuw.edu.pl

Materials:

Thioflavin T: was "UltraPure Grade" purchased from AnaSpec (USA). Two stock solutions were prepared: for standard ThT fluorescence assay the stock solution was of 3.14 mM whereas for lasing experiments was 9.4 mM in acetic acid and 78.4 mM in water.

FF: was purchased from Bachem (Switzerland). It was dissolved to 1.6 M in acetic acid.

KLVFFA: was purchased from FutureSynthesis (Poland) and was used as obtained (purity >99%). It was dissolved in water and in acetic acid to a concentration of 345 mM.

Human ABeta (1-42): peptide was purchased from GenScript and used as obtained (purity >95%). The protein was dissolved in water at a concentration of 55.4 mM. The solution was incubated at 37°C for 10 h. Aggregation was monitored by the ThT fluorescence assay (the results are shown in Fig. 1(e)). For lasing experiments the 4μL of protein concentrate and 2 μL of ThT stock were mixed and drop casted on the one cavity mirror. Then second cavity mirror was put on top. Sandwiched gain medium (ThT - ABeta (1-42)) between two mirrors was mounted in a heating holder. The initial measurement was done in room conditions and the rest measurements were performed at 37°C over time.

Methods:

fluorescence spectroscopy: Fluorescence measurements were performed using the Horiba QuantaMaster 8075-11 spectrofluorometer. Emission spectra were obtained by the exciting sample solutions in 1x1 cm quartz cuvettes with integrated xenon lamp. Both excitation and emission slits were set to 2 nm, and the spectra were corrected for detector sensitivity.

Lasing spectroscopy: A femtosecond pulse was generated at a 0.5 kHz repetition rate, providing pulse energy of 400 μJ at 430 nm through frequency doubling in a BBO crystal from an optical parametric amplifier (Orpheus by Light Conversion) pumped by a femtosecond amplifier (Carbide by Light Conversion).

The mirrors used for cavities had nearly 100% transmission at 400-450 nm. The reflectance in range 470-570 nm was around 95-99%, centered at 520 - 530 nm.

The lasing signal from the gain medium in cavities was collected parallel to the direction of the excitation beam. A filter was removing excitation >470 nm and only the lasing signal from the sample was reaching the detector. Lasing was collected with a ProEM Excelon camera from Teledyne Princton Instruments

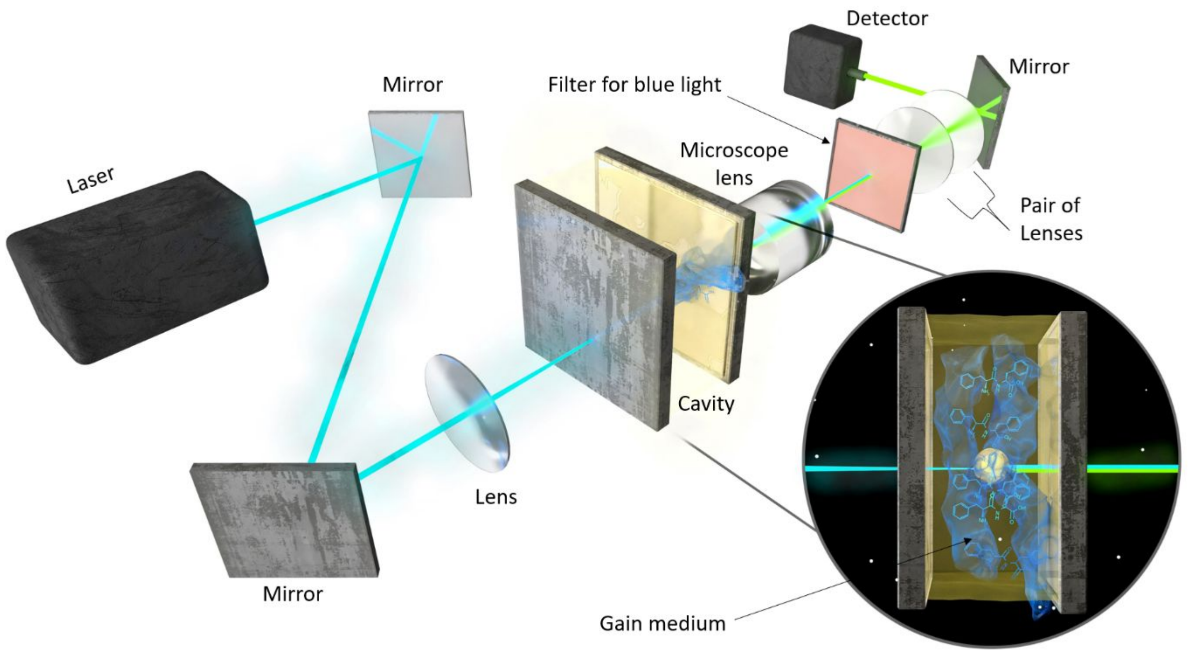

Fig. S1 *Schematic representation of the experimental lasing setup with enlarged cavity containing a gain medium which is diphenylalanine (FF) doped with ThT.*

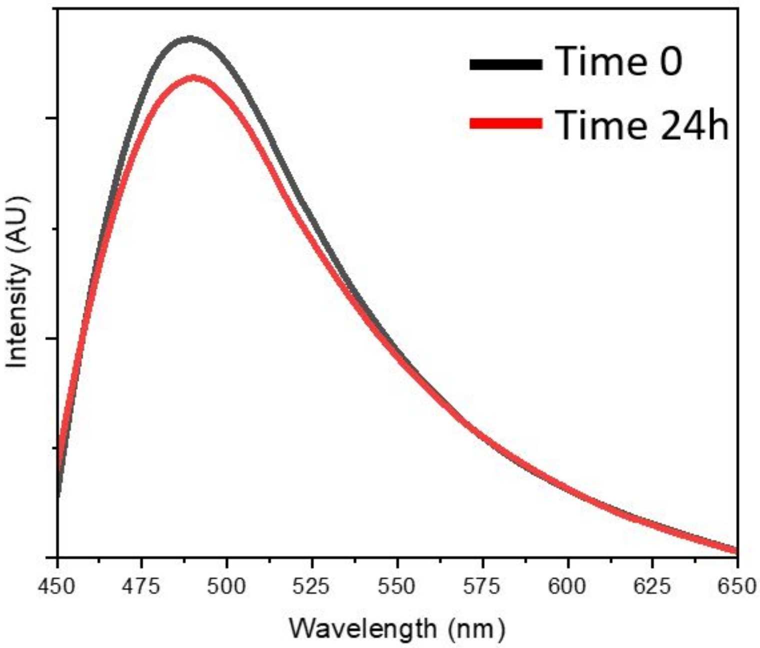

Fig. S2 Fluorescence spectra of FF measured in freshly dissolved sample (black line) and after 24h incubation (red line).

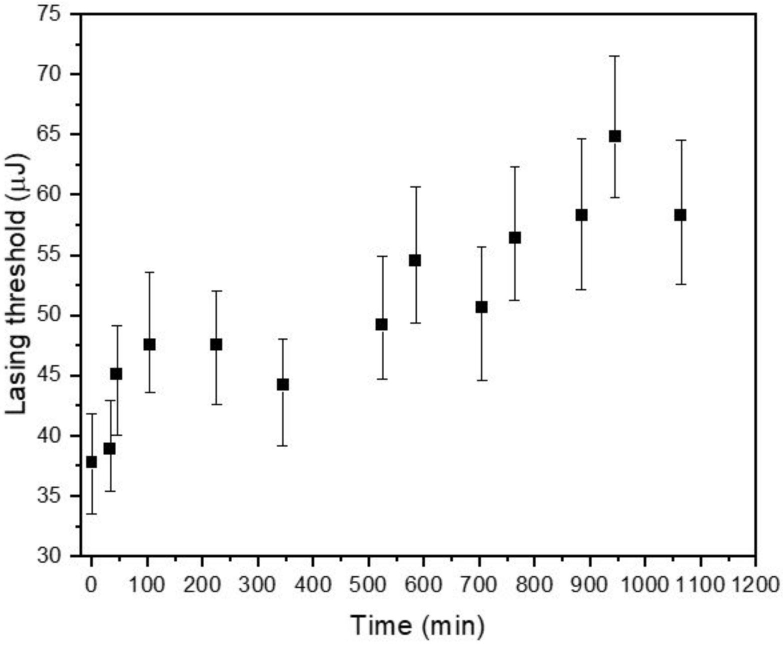

Fig. S3 Lasing thresholds measured over time in ThT mixed with LVEALYL dissolved at pH12, C_{ThT} = *26.1 mM,* $C_{LVEALYL}$ = *150 mM*
